# Ethyl 3-amino-4*H*-thieno[2,3-*b*]pyridine-2-carboxyl­ate

**DOI:** 10.1107/S1600536808039974

**Published:** 2008-12-03

**Authors:** Ren-lin Zheng, Wen-qin Zhang, Luo-Ting Yu, Sheng-Yong Yang, Li Yang

**Affiliations:** a State Key Laboratory of Biotherapy and Cancer Center, West China Hospital, West China Medical School, Sichuan University, Chengdu 610041, People’s Republic of China

## Abstract

The mol­ecule of the title compound, C_10_H_10_N_2_O_2_S, is essentially planar, except for the ethyl group, which is twisted away from the carboxyl plane by −90.5 (3)°. In the crystal structure, mol­ecules are linked into a zigzag sheet propagating along the *b* axis by inter­molecular N—H⋯O and N—H⋯N hydrogen bonds.

## Related literature

For general background, see: Litvinov *et al.* (2005 ▶).
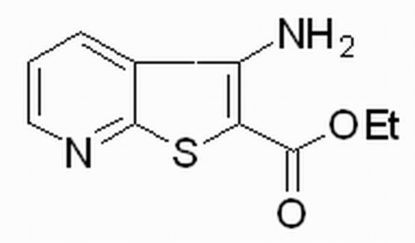


## Experimental

### 

#### Crystal data

C_10_H_10_N_2_O_2_S
*M* *_r_* = 222.26
Monoclinic, 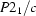

*a* = 6.657 (4) Å
*b* = 13.891 (4) Å
*c* = 10.902 (4) Å
β = 91.64 (4)°
*V* = 1007.8 (8) Å^3^
*Z* = 4
Mo *K*α radiation
μ = 0.30 mm^−1^
*T* = 292 (2) K
0.60 × 0.46 × 0.42 mm

#### Data collection

Enraf–Nonius CAD-4 diffractometer
Absorption correction: spherical (Dwiggins, 1975 ▶) *T*_min_ = 0.840, *T*_max_ = 0.884
1978 measured reflections
1864 independent reflections
1515 reflections with *I* > 2σ(*I*)
*R*_int_ = 0.008
3 standard reflections every 150 reflections intensity decay: 0.6%

#### Refinement

*R*[*F* ^2^ > 2σ(*F* ^2^)] = 0.040
*wR*(*F* ^2^) = 0.118
*S* = 1.14
1864 reflections
145 parameters
H atoms treated by a mixture of independent and constrained refinement
Δρ_max_ = 0.26 e Å^−3^
Δρ_min_ = −0.38 e Å^−3^

### 

Data collection: *DIFRAC* (Gabe & White, 1993 ▶); cell refinement: *DIFRAC*; data reduction: *NRCVAX* (Gabe *et al.*, 1989 ▶); program(s) used to solve structure: *SHELXS97* (Sheldrick, 2008 ▶); program(s) used to refine structure: *SHELXL97* (Sheldrick, 2008 ▶); molecular graphics: *ORTEP-3* (Farrugia, 1997 ▶); software used to prepare material for publication: *SHELXL97*.

## Supplementary Material

Crystal structure: contains datablocks global, I. DOI: 10.1107/S1600536808039974/ci2735sup1.cif

Structure factors: contains datablocks I. DOI: 10.1107/S1600536808039974/ci2735Isup2.hkl

Additional supplementary materials:  crystallographic information; 3D view; checkCIF report

## Figures and Tables

**Table 1:** Hydrogen-bond geometry (Å, °)

*D*—H⋯*A*	*D*—H	H⋯*A*	*D*⋯*A*	*D*—H⋯*A*
N2—H1N2⋯O2	0.84 (2)	2.26 (2)	2.848 (3)	127 (2)
N2—H1N2⋯O2^i^	0.84 (2)	2.38 (3)	3.067 (3)	139 (2)
N2—H2N2⋯N1^ii^	0.81 (3)	2.38 (3)	3.118 (3)	152 (3)

Symmetry codes: (i) 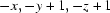; (ii) 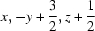.

**Fig. 1.**
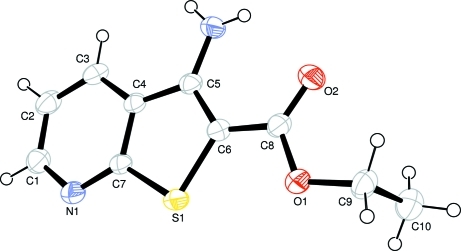
The molecular structure of the title compound, with displacement ellipsoids drawn at the 30% probability level.

**Fig. 2.**
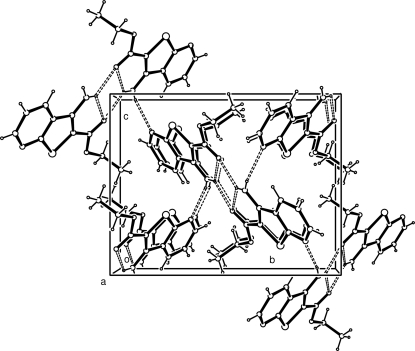
A packing diagram of the title compound. Intermolecular hydrogen bonds are shown as dashed open lines.

## supplementary crystallographic information

### Comment

Thieno[2,3-*b*]pyridine derivatives are of great importance owing to their wide biological properties (Litvinov *et al.*,2005). The title compound is one of the key intermediates in our synthetic investigations of antitumor drugs. We report here its crystal structure.

The thieno[2,3-*b*]pyridine ring system of the title molecule (Fig.1) is essentially planar. The amino group and the carbonyl group are nearly coplanar with the heterocyclic ring system. The ethyl group is twisted perpendicular to the remaining part of the molecule [C8—O1—C9—C10 = -90.5 (3)°].

In the crystal structure, the molecules are linked into a zigzag sheet propagating along the *b* axis by intermolecular N—H···O and N—H···N hydrogen bonds (Fig. 2).

### Experimental

A mixture of 2-chloro-3-cyanopyridine (3.3 g, 0.023 mol), ethyl 2-mercaptoacetate (3.62 g, 0.03 mol), sodium carbonate (2.65 g, 0.025 mol) and anhydrous ethanol (12.0 ml) was heated for 4.5 h under reflux. The reaction mixture was cooled to ambient temperature and added to water (150 ml). The resultant precipitate was stirred for 45 min and then filtered. The filter cake was washed with two portions of water (25 ml) and dried to yield the title compound as a yellow solid (5.032 g, 95.1% yield). Single crystals suitable for X-ray analysis were obtained by slow evaporation of a tetrahydrofuran solution.

### Refinement

H atoms of the amino group were located in a difference map and refined freely. The reminaing H atoms were positioned geometrically (C—H = 0.93–0.97 Å) and refined using a riding model, with *U*_iso_(H) = 1.2–1.5*U*_eq_(C).

### Crystal data

**Table:**

C_10_H_10_N_2_O_2_S	*F*(000) = 464
*M**_r_* = 222.26	*D*_x_ = 1.465 Mg m^−^^3^
Monoclinic, *P*2_1_/*c*	Mo *K*α radiation, λ = 0.71073 Å
Hall symbol: -P 2ybc	Cell parameters from 22 reflections
*a* = 6.657 (4) Å	θ = 4.3–5.7°
*b* = 13.891 (4) Å	µ = 0.30 mm^−^^1^
*c* = 10.902 (4) Å	*T* = 292 K
β = 91.64 (4)°	Block, colourless
*V* = 1007.8 (8) Å^3^	0.60 × 0.46 × 0.42 mm
*Z* = 4	

### Data collection

**Table:**

Enraf–Nonius CAD-4 diffractometer	1515 reflections with *I* > 2σ(*I*)
Radiation source: fine-focus sealed tube	*R*_int_ = 0.008
graphite	θ_max_ = 25.5°, θ_min_ = 2.4°
ω/2θ scans	*h* = −8→8
Absorption correction: for a sphere (Dwiggins, 1975)	*k* = 0→16
*T*_min_ = 0.840, *T*_max_ = 0.884	*l* = −6→13
1978 measured reflections	3 standard reflections every 150 reflections
1864 independent reflections	intensity decay: 0.6%

### Refinement

**Table:**

Refinement on *F*^2^	Primary atom site location: structure-invariant direct methods
Least-squares matrix: full	Secondary atom site location: difference Fourier map
*R*[*F*^2^ > 2σ(*F*^2^)] = 0.040	Hydrogen site location: mixed
*wR*(*F*^2^) = 0.118	H atoms treated by a mixture of independent and constrained refinement
*S* = 1.14	*w* = 1/[σ^2^(*F*_o_^2^) + (0.0562*P*)^2^ + 0.4962*P*] where *P* = (*F*_o_^2^ + 2*F*_c_^2^)/3
1864 reflections	(Δ/σ)_max_ = 0.001
145 parameters	Δρ_max_ = 0.26 e Å^−^^3^
0 restraints	Δρ_min_ = −0.37 e Å^−^^3^

### Special details

**Table:**

Geometry. All e.s.d.'s (except the e.s.d. in the dihedral angle between two l.s. planes) are estimated using the full covariance matrix. The cell e.s.d.'s are taken into account individually in the estimation of e.s.d.'s in distances, angles and torsion angles; correlations between e.s.d.'s in cell parameters are only used when they are defined by crystal symmetry. An approximate (isotropic) treatment of cell e.s.d.'s is used for estimating e.s.d.'s involving l.s. planes.
Refinement. Refinement of *F*^2^ against ALL reflections. The weighted *R*-factor *wR* and goodness of fit *S* are based on *F*^2^, conventional *R*-factors *R* are based on *F*, with *F* set to zero for negative *F*^2^. The threshold expression of *F*^2^ > σ(*F*^2^) is used only for calculating *R*-factors(gt) *etc*. and is not relevant to the choice of reflections for refinement. *R*-factors based on *F*^2^ are statistically about twice as large as those based on *F*, and *R*- factors based on ALL data will be even larger.

### Fractional atomic coordinates and isotropic or equivalent isotropic displacement parameters (Å^2^)

**Table:**

	*x*	*y*	*z*	*U*_iso_*/*U*_eq_	
S1	0.20405 (9)	0.73591 (5)	0.16994 (5)	0.0405 (2)	
O1	−0.1351 (2)	0.60921 (12)	0.17935 (15)	0.0442 (4)	
O2	−0.0551 (3)	0.52930 (13)	0.35384 (16)	0.0487 (5)	
N1	0.5470 (3)	0.83735 (15)	0.20206 (18)	0.0416 (5)	
N2	0.3157 (4)	0.58481 (17)	0.46991 (19)	0.0429 (5)	
H1N2	0.215 (4)	0.5502 (18)	0.481 (2)	0.032 (7)*	
H2N2	0.407 (4)	0.593 (2)	0.519 (3)	0.049 (8)*	
C1	0.7139 (4)	0.8539 (2)	0.2686 (2)	0.0461 (6)	
H1	0.8010	0.9013	0.2419	0.055*	
C2	0.7661 (4)	0.8045 (2)	0.3756 (2)	0.0468 (6)	
H2	0.8851	0.8192	0.4184	0.056*	
C3	0.6422 (4)	0.73450 (18)	0.4178 (2)	0.0394 (6)	
H3	0.6751	0.7009	0.4894	0.047*	
C4	0.4659 (3)	0.71448 (16)	0.35153 (19)	0.0326 (5)	
C5	0.3088 (3)	0.64550 (16)	0.3736 (2)	0.0333 (5)	
C6	0.1607 (3)	0.64942 (17)	0.2830 (2)	0.0348 (5)	
C7	0.4275 (3)	0.76841 (17)	0.2448 (2)	0.0344 (5)	
C8	−0.0161 (4)	0.59041 (17)	0.2784 (2)	0.0363 (5)	
C9	−0.3115 (4)	0.5493 (2)	0.1610 (2)	0.0457 (6)	
H9A	−0.4178	0.5864	0.1209	0.055*	
H9B	−0.3587	0.5280	0.2398	0.055*	
C10	−0.2633 (4)	0.4637 (2)	0.0840 (3)	0.0527 (7)	
H10A	−0.2104	0.4849	0.0076	0.079*	
H10B	−0.3832	0.4268	0.0684	0.079*	
H10C	−0.1653	0.4245	0.1267	0.079*	

### Atomic displacement parameters (Å^2^)

**Table:**

	*U*^11^	*U*^22^	*U*^33^	*U*^12^	*U*^13^	*U*^23^
S1	0.0453 (4)	0.0474 (4)	0.0286 (3)	−0.0085 (3)	−0.0057 (2)	0.0056 (2)
O1	0.0454 (10)	0.0502 (10)	0.0366 (9)	−0.0120 (8)	−0.0092 (7)	0.0033 (8)
O2	0.0504 (11)	0.0543 (11)	0.0411 (10)	−0.0142 (8)	−0.0030 (8)	0.0107 (8)
N1	0.0478 (12)	0.0464 (12)	0.0307 (10)	−0.0095 (9)	0.0028 (9)	0.0007 (9)
N2	0.0456 (13)	0.0488 (13)	0.0338 (11)	−0.0089 (11)	−0.0060 (10)	0.0094 (9)
C1	0.0465 (14)	0.0514 (16)	0.0404 (14)	−0.0145 (12)	0.0050 (11)	−0.0037 (12)
C2	0.0390 (14)	0.0640 (17)	0.0372 (13)	−0.0085 (12)	−0.0022 (10)	−0.0084 (12)
C3	0.0399 (13)	0.0513 (14)	0.0268 (11)	−0.0002 (11)	−0.0007 (9)	−0.0036 (10)
C4	0.0371 (12)	0.0358 (12)	0.0250 (11)	−0.0001 (9)	0.0024 (9)	−0.0048 (9)
C5	0.0380 (12)	0.0364 (12)	0.0258 (11)	0.0013 (9)	0.0024 (9)	−0.0026 (9)
C6	0.0396 (13)	0.0372 (12)	0.0277 (11)	−0.0029 (10)	−0.0002 (9)	0.0008 (9)
C7	0.0404 (13)	0.0387 (12)	0.0242 (10)	−0.0020 (10)	0.0024 (9)	−0.0039 (9)
C8	0.0396 (13)	0.0390 (13)	0.0303 (12)	−0.0014 (10)	−0.0012 (10)	−0.0023 (10)
C9	0.0386 (14)	0.0545 (16)	0.0435 (14)	−0.0059 (11)	−0.0068 (11)	−0.0061 (12)
C10	0.0517 (16)	0.0523 (16)	0.0540 (16)	−0.0050 (13)	0.0001 (13)	−0.0078 (13)

### Geometric parameters (Å, °)

**Table:**

S1—C7	1.736 (3)	C2—H2	0.93
S1—C6	1.752 (2)	C3—C4	1.389 (3)
O1—C8	1.347 (3)	C3—H3	0.93
O1—C9	1.448 (3)	C4—C7	1.401 (3)
O2—C8	1.215 (3)	C4—C5	1.444 (3)
N1—C1	1.330 (3)	C5—C6	1.376 (3)
N1—C7	1.337 (3)	C6—C8	1.434 (3)
N2—C5	1.346 (3)	C9—C10	1.495 (4)
N2—H1N2	0.84 (2)	C9—H9A	0.97
N2—H2N2	0.81 (3)	C9—H9B	0.97
C1—C2	1.389 (4)	C10—H10A	0.96
C1—H1	0.93	C10—H10B	0.96
C2—C3	1.364 (4)	C10—H10C	0.96
			
C7—S1—C6	90.20 (11)	C5—C6—C8	125.0 (2)
C8—O1—C9	117.08 (19)	C5—C6—S1	113.77 (17)
C1—N1—C7	115.4 (2)	C8—C6—S1	121.27 (17)
C5—N2—H1N2	117.9 (17)	N1—C7—C4	125.2 (2)
C5—N2—H2N2	116 (2)	N1—C7—S1	122.16 (18)
H1N2—N2—H2N2	125 (3)	C4—C7—S1	112.63 (17)
N1—C1—C2	123.9 (2)	O2—C8—O1	123.1 (2)
N1—C1—H1	118.0	O2—C8—C6	124.5 (2)
C2—C1—H1	118.0	O1—C8—C6	112.4 (2)
C3—C2—C1	119.8 (2)	O1—C9—C10	110.4 (2)
C3—C2—H2	120.1	O1—C9—H9A	109.6
C1—C2—H2	120.1	C10—C9—H9A	109.6
C2—C3—C4	118.5 (2)	O1—C9—H9B	109.6
C2—C3—H3	120.7	C10—C9—H9B	109.6
C4—C3—H3	120.7	H9A—C9—H9B	108.1
C3—C4—C7	117.1 (2)	C9—C10—H10A	109.5
C3—C4—C5	130.7 (2)	C9—C10—H10B	109.5
C7—C4—C5	112.2 (2)	H10A—C10—H10B	109.5
N2—C5—C6	126.3 (2)	C9—C10—H10C	109.5
N2—C5—C4	122.5 (2)	H10A—C10—H10C	109.5
C6—C5—C4	111.2 (2)	H10B—C10—H10C	109.5
			
C7—N1—C1—C2	0.0 (4)	C1—N1—C7—C4	0.0 (4)
N1—C1—C2—C3	−0.1 (4)	C1—N1—C7—S1	−179.49 (18)
C1—C2—C3—C4	0.1 (4)	C3—C4—C7—N1	0.0 (4)
C2—C3—C4—C7	0.0 (3)	C5—C4—C7—N1	179.9 (2)
C2—C3—C4—C5	−180.0 (2)	C3—C4—C7—S1	179.53 (17)
C3—C4—C5—N2	−1.1 (4)	C5—C4—C7—S1	−0.5 (2)
C7—C4—C5—N2	178.9 (2)	C6—S1—C7—N1	179.8 (2)
C3—C4—C5—C6	−179.5 (2)	C6—S1—C7—C4	0.22 (18)
C7—C4—C5—C6	0.6 (3)	C9—O1—C8—O2	−3.2 (3)
N2—C5—C6—C8	1.9 (4)	C9—O1—C8—C6	176.0 (2)
C4—C5—C6—C8	−179.8 (2)	C5—C6—C8—O2	−0.6 (4)
N2—C5—C6—S1	−178.65 (19)	S1—C6—C8—O2	−179.94 (19)
C4—C5—C6—S1	−0.4 (3)	C5—C6—C8—O1	−179.8 (2)
C7—S1—C6—C5	0.12 (19)	S1—C6—C8—O1	0.9 (3)
C7—S1—C6—C8	179.6 (2)	C8—O1—C9—C10	−90.5 (3)

### Hydrogen-bond geometry (Å, °)

**Table:**

*D*—H···*A*	*D*—H	H···*A*	*D*···*A*	*D*—H···*A*
N2—H1N2···O2	0.84 (2)	2.26 (2)	2.848 (3)	127 (2)
N2—H1N2···O2^i^	0.84 (2)	2.38 (3)	3.067 (3)	139 (2)
N2—H2N2···N1^ii^	0.81 (3)	2.38 (3)	3.118 (3)	152 (3)

Symmetry codes: (i) −*x*, −*y*+1, −*z*+1; (ii) *x*, −*y*+3/2, *z*+1/2.
